# Insomnia and Hypertension: Importance of Objective Phenotyping and Comorbidity with Sleep Apnea

**DOI:** 10.1007/s11906-026-01365-8

**Published:** 2026-02-09

**Authors:** Nikolaos Athanasiou, Alexandros N. Vgontzas, Julio Fernandez-Mendoza

**Affiliations:** 1 Penn State Health Sleep Research and Treatment Center, Pennsylvania State University, College of Medicine, Hershey, PA USA; 2 Department of Psychiatry & Behavioral Health, Pennsylvania State University, College of Medicine, 500 University Drive, Hershey, PA 17033 USA

**Keywords:** Insomnia, Phenotypes, Sleep apnea, COMISA, Hypertension, Elevated blood pressure

## Abstract

**Purpose of Review:**

Provide a synthesis of research from the past five years examining the relationship between insomnia, its phenotypes, and comorbidity with sleep apnea (COMISA), with hypertension. Offer a critical evaluation of current evidence and outline future research directions.

**Recent Findings:**

Meta-analytic evidence indicates that the risk of hypertension is higher in patients with insomnia disorder compared to those with insomnia symptoms, with the highest risk observed in the insomnia with objective short sleep duration phenotype. COMISA appears to further amplify this risk, although the evidence is limited to individual studies with no meta-analyses available. There is a clear need for clinical trials to confirm these associations and guide the development of more effective therapeutic strategies.

**Summary:**

Insomnia should be part of the diagnostic assessment of patients with hypertension. Objective sleep duration can serve as a biomarker, alongside respiratory indices, to guide therapeutic decision-making and optimize patient management.

## Introduction

Hypertension affects almost half of the adult population in the United States and represents a major risk factor for cardiovascular disease [1–3]. Insomnia, one of the most prevalent sleep disorders in the general population, has also been implicated as a potential risk factor for hypertension [4]. However, due to heterogeneity among studies regarding its definition, it has remained elusive understanding who among those with insomnia have a significant risk of hypertension. Insomnia has often been reported either as insomnia symptoms —affecting 30–40% of the general population [5, 6]— or as insomnia disorder, with an estimated 10–15% meeting the diagnostic criteria for chronic insomnia disorder [5–7]. Insomnia symptoms consist of self-reported difficulties falling asleep at bedtime (difficulty initiating sleep, DIS), staying asleep during the sleep period (difficulty maintaining sleep, DMS), or waking up too early in the morning before desired and being unable to resume sleep (early morning awakening, EMA) [5, 6]. The chronic form of insomnia disorder, the most common presentation encountered in clinical settings, is defined by the presence of at least one of these nighttime symptoms, accompanied by notable daytime impairment with social, occupational, educational, academic, and behavioral distress or impairment, despite adequate sleep opportunity and suitable circumstances for sleep [7]. To fulfill diagnostic criteria, symptoms must occur at least three nights per week, persist for a minimum duration of three months, and cannot be better explained by another sleep, medical, or psychiatric disorder, or by medication/substance use [7].

The etiology and pathophysiology of insomnia are multifactorial, involving a complex interplay of genetic, neurobiological, behavioral, and cognitive-emotional factors [8]. Dysregulation of the stress response system, particularly hyperactivity of the hypothalamic–pituitary–adrenal (HPA) axis and sympathetic nervous system, has been identified as a key underlying mechanism contributing to its persistence and clinical manifestation; however, this dysregulation has been primarily found in the insomnia short sleep duration phenotype (ISSD) [8–10]. Based on such early observation and in an effort to phenotypically distinguish patients with insomnia and identify those with greater clinical severity and cardiovascular risk, Vgontzas et al. employed objective total sleep time (TST) measured by polysomnography (PSG) as a useful marker to estimate cardiovascular risk among those with insomnia [10–12]. Findings indicated that the ISSD phenotype is associated with the most adverse cardiovascular and metabolic risk profile, as well as with emerging evidence linking this phenotype to alterations in the gut microbiome and elevated blood pressure (BP) [10–15]. In contrast, the insomnia with normal sleep duration (INSD) phenotype did not show a significant risk of cardiovascular or metabolic sequelae in these studies.

Sleep disordered breathing, especially obstructive sleep apnea (OSA), affects about 10–20% of the general population, with robust evidence supporting its role as a recognized risk factor for high blood pressure and clinical hypertension [3, 16]. The coexistence of OSA with insomnia disorder, commonly referred to as comorbid insomnia and sleep apnea (COMISA), is frequently observed in clinical practice, with prevalence rates in cohorts of clinical patients between 18% and 42% [17, 18]. Approximately 30–50% of individuals with OSA experience clinically relevant insomnia symptoms, while 30–40% of those diagnosed with insomnia disorder fulfill diagnostic criteria for OSA [17, 18]. However, the literature linking this specific clinical entity to hypertension risk and elevated blood pressure remains limited.

This review aims to summarize the evidence accrued over the past five years addressing insomnia and its clinical phenotypes in relation to hypertension. It also seeks to elucidate the current understanding of COMISA, a condition that has garnered increasing research attention in recent years and represents a challenging entity from both pathophysiological and therapeutic perspectives. Finally, it aims to outline future research directions necessary to advance knowledge in this field, particularly in an era of rapid technological progress and the growing implementation of personalized medicine.

## Clinical Presentation of Insomnia: Association with Hypertension

Despite more than four decades of research and clinical experience, there remains considerable variability in how insomnia is defined and assessed [19]. Some studies rely on one or two questions to determine an individual’s insomnia status, whereas others use formal diagnostic criteria to classify it as a disorder. Even within standardized nosology, there are notable differences in diagnostic approaches, reflecting a historical divide between “lumping” versus “splitting” strategies [19]. The term insomnia is often broadly used to refer to the nighttime symptoms of difficulty sleeping. However, while this broad definition is simple and can be useful under certain circumstances, it fails to capture the full spectrum of insomnia, including the specific symptom, severity, frequency, duration, and functional consequences. Thus, despite the long-standing variability in definitions surrounding insomnia disorder, and the historical view of insomnia as a secondary manifestation of another condition, it has been recognized as an independent disorder in its own right [19, 20]. This paradigm shift has opened new avenues for both research and therapeutic approaches. However, as previously mentioned, insomnia continues to appear in the literature defined as a symptom more than as a disorder.

Although the association between insomnia and hypertension was first noted in the 1970s, it was initially considered secondary to hypertension, which resulted in limited investigation of insomnia’s potential independent association with clinical or subclinical indicators of elevated or dysregulated BP in the following decades [21–25]. Meta-analyses examining the association between insomnia and hypertension over the past five years have noted that most studies relied on self-reported insomnia symptoms, reflecting practical constraints, as many were based on large population or epidemiologic cohorts [26–29].

The studies outlined in Table 1 support that adults with self-reported insomnia symptoms have a 1.05- to 1.21-fold increased risk for incident hypertension and about 1.41-fold risk of prevalent hypertension [26–28]. Moreover, these meta-analytic data indicate that individuals with insomnia exhibit a blunted nocturnal decline in both systolic and diastolic blood pressure, suggesting an attenuated dipping pattern [29]. Notably, two meta-analyses consistently found that, among all insomnia symptoms, DIS was the only one not associated with a significantly increased risk of hypertension, while the highest risk was observed among individuals reporting DMS [26, 27]. Moreover, one of these meta-analyses showed that heterogeneity emerged when the analyses were stratified by ethnicity [27]. The association of insomnia with hypertension was statistically significant in the European population (RR = 1.08, 95% CI: 1.02–1.14) and Australian population (RR = 1.15; 95%CI: 1.04–1.28), whereas it did not reach statistical significance in the Asian (RR = 1.54, 95% CI: 0.98–2.40) or American population (RR = 1.21, 95% CI: 0.89–1.65) despite the greater relative risk observed. Investigators interpreted these findings as potentially explained by differences in prevalence rates and heterogeneity in factors that widen the confidence intervals in the Asian and American populations, such as smoking, obesity, physical activity level, alcohol use and/or socioeconomic status [27]. Finally, another indicator of good cardiovascular function is BP dipping, which reflects the normal nocturnal decline in blood pressure (10–20%) [30]. In the meta-analysis by Maiolino et al., it was found that patients with insomnia exhibit impaired systolic and diastolic BP dipping compared to controls [29].

**Table 1:** Meta-analyses on the association between insomnia and its phenotypes with hypertension and blood pressure dipping

First author, year (studies’ design)	*N* (# of studies)	Insomnia Definition	Outcome	Findings
Meng, 2013 (Longitudinal) [26]	42,636 (7)	Difficulty initiating sleep Difficulty maintaining sleep Early morning awakening Insomnia symptoms	HTN	RR = 1.17 RR = 1.20* RR = 1.14* RR = 1.05*
Li L, 2021 (Longitudinal) [27]	395,641 (14)	Difficulty initiating sleep Difficulty maintaining sleep Early morning awakening Insomnia symptoms	HTN	RR = 1.14 RR = 1.27* RR = 1.14* RR = 1.21*
Zhang, 2021 (Cross-sectional) [28]	151,299 (12)	Insomnia symptoms	HTN	OR = 1.41*
Maiolino, 2021 (Cross-sectional) [29]	924 (4)	Insomnia symptoms or disorder	SBPD DBPD	Percent = -2.00* Percent = -1.58*
Johnson, 2021 (Cross-sectional) [37]	3,034 (7)	Insomnia disorder PSG for < 6 h of TST	HTN	RR = 1.54*
Dai, 2024 (Cross-sectional) [38]	5914 (6)	Insomnia disorder PSG for < 6 h of TST	HTN	OR = 2.67*
Dai, 2024 (Longitudinal) [38]	1963 (2)	Insomnia disorder PSG for < 6 h of TST	HTN	RR = 1.95*

Note: at the time of this review there were no published meta-analysis on the association of (COMISA) and hypertension

DBPD = diastolic blood pressure dipping. HTN = hypertension. OR = odds ratio. RR = relative risk. SBPD = systolic blood pressure dipping

* = Statistically significant

Although these meta-analyses are based on studies that account for a wide range of confounding factors, they did not specifically assess the impact of chronic insomnia disorder or incorporate PSG, limiting their capacity to control for OSA or to specifically assess associations with objectively-measured insomnia phenotypes.

## Phenotypes of Insomnia: Association with Hypertension

Within the broader effort over the past two decades to conceptualize insomnia as an independent clinical entity, the need has emerged to incorporate objective measures to more accurately assess and phenotype patients. The Penn State group was the first to propose that objective features of sleep disturbance, such as objectively-measured sleep duration, may serve as indicators of the biological severity of insomnia, as short sleep duration had been associated with hyperactivity of the HPA axis in individuals with insomnia disorder [9, 10]. In continuation of their neurobiological observations, the same group showed that the ISSD phenotype was associated with 5.1-fold increased odds of prevalent hypertension and 3.7-fold increased odds of incident hypertension [11, 12]. In contrast, the INSD phenotype was not found to significantly increase the risk of either prevalent (OR = 1.31; 95%CI: 0.70–2.46) or incident (OR = 0.85 95%CI: 0.30–2.40) hypertension [11, 12]. An important aspect of these studies to note is that they distinguished individuals with chronic insomnia from those with insomnia symptoms, thereby strengthening the evidence on the relationship between the disorder and hypertension. The findings were also important as they were derived after adjusting for multiple demographic (e.g., age, sex, race/ethnicity), clinical (e.g., OSA, diabetes, depression), and behavioral (e.g., smoking, alcohol, caffeine) factors, thereby paving the way for further investigation by other research groups, which subsequently replicated them in other cohort studies [31–36]. Indeed, two meta-analyses conducted over the past five years have examined the association of the ISSD and INSD phenotypes with hypertension, including cross-sectional and longitudinal studies [37, 38]. Johnson et al. showed that individuals with the ISSD phenotype had a 1.54-fold risk of prevalent hypertension compared with those with the INSD phenotype [37]. No statistically significant differences in hypertension risk were observed between individuals with ISSD and those with objective short sleep duration without insomnia, leading authors to conclude that the cardiometabolic burden attributed to ISSD may largely reflect the effects of short sleep itself [37]. However, methodological issues may have influenced the validity of the findings in this meta-analysis, such as relying on descriptive unadjusted data instead of multivariable-adjusted estimates (e.g., OR, RR) adequately controlled for confounders, absence of a reference group (i.e., good sleepers with normal sleep duration), and combining data from cohort studies of randomly-selected population-based samples with experimental studies that had specific inclusion/exclusion criteria, or including data from studies not specifically testing the association of insomnia phenotypes with hypertension. To address these methodological limitations, a subsequent meta-analysis by Dai et al. included reference groups (both good sleepers and INSD) and relied on multivariable-adjusted data (i.e., odds ratios, hazard ratios, and relative risks) from cohort studies testing the association of these insomnia phenotypes with hypertension, incorporating a more rigorous adjustment for potential confounders [38]. Multivariable-adjusted estimates derived from six cross-sectional studies with 5,914 participants and two longitudinal studies with 1,963 participants showed that the ISSD phenotype was associated with a 2.67-fold increased risk of prevalent hypertension and 1.95-fold increased risk of incident hypertension compared with good sleepers (i.e., individuals without insomnia and normal sleep duration). In addition, the ISSD phenotype was associated with a 1.94-fold increased risk of prevalent hypertension and a 2.07-fold increased risk of incident hypertension compared to the INSD phenotype. Finally, compared to good sleepers, those with objective short sleep duration without insomnia were not associated with significantly increased odds of either prevalent (OR = 1.21, 95%CI: 0.84–1.75) or incident (OR = 0.97, 95%CI: 0.81–1.17) hypertension. Collectively, these findings led authors to conclude that ISSD is the insomnia phenotype carrying true hypertension risk and that it’s cardiometabolic burden does not simply reflect the effects of short sleep itself [38].

Alternative phenotyping approaches have also been proposed in the literature, such as following a similar conceptual sleep duration framework as above but relying on subjective rather than objective measures to define the ISSD phenotype; these studies have, however, yield imprecise estimates or nonsignificant associations [31, 34, 39, 40]. Another study adopted an alternative approach to objective phenotypic classification based on the multiple sleep latency test (MSLT). Li et al. showed that individuals with chronic insomnia and an MSLT-measured sleep onset latency greater than 14 min had 3-fold higher odds of prevalent hypertension compared with normal sleepers exhibiting an MSLT-measured sleep onset latency of 14 min or less [41]. Moreover, when MSLT sleep onset latency exceeded 17 min, the odds of hypertension increased approximately 4-fold, consistent with the dose-response association with systolic and diastolic BP levels observed in the same study [41].

## Comorbidity of Insomnia and Sleep Apnea: Association with Hypertension

Although the co-occurrence of insomnia and sleep apnea was first documented in 1973, research on this comorbidity has only flourished in recent years [18, 42]. As a result, there are no meta-analyses or systematic reviews yet addressing this clinical entity comprehensively, and particularly in relation to hypertension. This limited attention may, at least in part, stem from the long-standing view of insomnia as a “secondary” condition, one precipitated and maintained by the assumed “primary” disorder (OSA) rather than as an independent and coexisting clinical phenomenon [20]. Thus, while heterogeneity has been described above regarding the definition and phenotypes of insomnia and the variability across studies, ΟSA may further complicate the interpretation of findings, creating a complex and intertwined network of potential interactions between these two or three entities. This complexity also arises from the lack of uniformity in the apnea/hypopnea index (AHI) cut-off values applied across studies, as well as from differences in patients’ phenotypic characteristics and underlying pathophysiological mechanisms (including anatomical factors, low arousal threshold, impaired upper airway dilator muscle function, and instability of ventilatory control) [43]. Nevertheless, it has been demonstrated that even mild-to-moderate forms of OSA can induce hypertension and inflammation, findings that are further supported by the studies discussed below that focus on COMISA and highlight the synergistic interaction between the two disorders that constitute this comorbidity [44–47].

Only few studies have been published on the association of COMISA with clinical hypertension and elevated BP, therefore, we will summarize herein the most significant ones. As shown in Table 2, population-based cohort studies using self-reported and clinical data have shown a significant relationship between COMISA and hypertension with estimated odds of prevalent hypertension ranging from 2- to 4-fold and estimated risk of incident hypertension of about 2-fold, depending on the definition used [48–52]. Specifically, a cross-sectional study with a wide age range (18–80 years) in Chinese adults with chronic insomnia (*N* = 860) showed that those with severe OSA (AHI > 30) had 3.68-old odds (95%CI: 1.47–9.21) of prevalent hypertension compared to those with chronic insomnia without OSA (AHI < 5), even after controlling for relevant covariates including obesity, sleepiness and heart disease [48]. However, since the authors did not include a control group without sleep disorders, it remained unclear how much of this association was due to the comorbidity or to OSA-alone. Lechat et al. reported that COMISA was associated with 2-fold risk (95% CI = 1.41–2.96) of prevalent hypertension compared to individuals without insomnia symptoms or OSA, although it remains unclear whether the association accounted for potential confounding factors [49]. Additionally, the study reported that OSA-alone (HR = 1.85, 95% CI 1.34–2.56) was associated with an increased risk of hypertension compared with the reference group, while the risk associated with insomnia symptoms-alone was not reported. Based on the descriptive data, the prevalence of hypertension in those with insomnia symptoms appeared comparable to that of the reference group (23% vs. 26%), suggesting no significant association between isolated insomnia symptoms and hypertension in this sample. A recent study in a middle-aged population (*N* = 3,832) showed that COMISA was associated with 1.9-fold (95%CI: 1.23–2.89) odds of uncontrolled hypertension compared to individuals without insomnia symptoms or OSA, even after adjusting for demographic, clinical and lifestyle factors [50]. A similar association was observed in the OSA-only group (OR = 1.31, 95%CI: 1.05–1.64) compared to the reference group, whereas no significant association was found for the insomnia symptoms-only group.

**Table 2:** Recent individual studies on the association between comorbid insomnia and sleep apnea (COMISA) with hypertension

First author, year (study design)	*N*	COMISA Definition	Outcome	Findings
Li Z, 2015 (Cross-sectional) [48]	860	Insomnia disorder PSG for AHI > 5	HTN	OR = 3.68*
Lechat, 2021 (Cross-sectional) [49]	1,115	Insomnia symptoms PSG for AHI ≥ 5	HTN	HR = 2.04*
Li X, 2021 (Longitudinal) [51]	6,965	Insomnia symptoms HSAT for AHI ≥ 5	HTN	OR = 2.09*
Pejovic, 2024 (Cross-sectional) [52]	101	Insomnia symptoms PSG for < 6 h of TST PSG or HSAT for 5 ≤ AHI < 30	HTN	OR = 3.88*
Kobayashi Frisk, 2025 (Cross-sectional) [50]	3,832	Insomnia Severity Index ≥ 15 HSAT for AHI > 10	HTN	OR = 1.88*

Note: at the time of this review there were no published systematic reviews or meta-analyses on the association of COMISA with hypertension

HR = hazard ratio. HSAT = home sleep apnea test. HTN = hypertension. OR = odds ratio. PSG = polysomnography

* = Statistically significant

There are two additional studies with unique methodological characteristics. One study had a longitudinal design and the other further phenotyped COMISA based on objective short sleep duration [51, 52]. In the study by Li X et al., which included a large cohort of U.S. Hispanic/Latine adults (*n* = 11,623) and provided longitudinal data on hypertension after 6 years of follow-up (final analytic sample *N* = 6,965), investigators showed that COMISA was associated with 2-fold (95% CI: 1.45–3.00) odds of incident hypertension compared to individuals without insomnia symptoms or OSA, even after full adjustment for a wide range of confounding factors [51]. Moreover, the OSA-only group was associated with 1.55-fold (95%CI: 1.14–2.09) odds of incident hypertension and the insomnia symptoms-only group with 1.37-fold (95%CI: 1.04–1.81) compared with the reference group. The other recent cross-sectional study in a clinical sample suggested that the higher prevalence of hypertension in COMISA patients appears to be primarily driven by the ISSD phenotype [52]. Specifically, the odds of prevalent hypertension were 3.9-fold (95% CI: 1.26–11.95) in patients with COMISA who slept less than 6 h (i.e., ISSD phenotype) compared to patients with COMISA who slept more than 6 h (i.e., INSD phenotype). Moreover, both systolic and diastolic BP levels were significantly higher in patients with COMISA and ISSD compared to patients with COMISA and INSD, even after adjustment for multiple demographic and clinical factors. This was the first study to introduce this insomnia phenotyping approach to investigating COMISA in relation to hypertension.

## Future Directions and Clinical Implications

Although OSA is widely recognized as a common cause of hypertension in clinical guidelines, insomnia disorder – despite its higher prevalence – has not gained the same status [1–4]. The present review does support that insomnia should be recognized as a modifiable risk factor for hypertension, and in particular for the ISSD phenotype, and emphasizes that insomnia should be considered as a potential factor for sleep consultation, examination, and treatment in the routine evaluation of a hypertensive patient.

However, there remains a need for randomized controlled trials to further strengthen this observation and lay the groundwork for a paradigm shift within the scientific community. In this direction, a unified definition and approach to insomnia disorder, along with improved patient assessment through standardized screening tools and thorough clinical evaluation should be implemented, even in large cohort studies [19]. Furthermore, measuring BP during patient evaluation and clearly defining hypertension stages based on objective measurements and current hypertension treatment status will contribute to a more holistic understanding compared with the self-reported diagnosis of hypertension often used in various studies [11, 31, 33–36] or the reliance on office BP levels rather than 24 h ambulatory monitoring [3]. Consequently, establishing a unified scientific language will enable the effective utilization of these findings to improve patient care.

The considerations mentioned above will also facilitate a more precise phenotypic characterization of patients, as evidence indicates that the ISSD phenotype represents the most pathophysiologically and medically severe subtype [9–15, 31]. Nevertheless, given the high prevalence of insomnia disorder in the general population, conducting PSG in all individuals would be financially impractical within current healthcare and insurance frameworks. In this context, and in an effort to establish a common scientific language both in sleep and circadian research, recommendations have been made for the use and evaluation of wearable devices, which are increasingly adopted in today’s era of technological advancement [53, 54]. The integration of such technologies is expected to mitigate methodological heterogeneity and enhance the rigor, reproducibility, and overall reliability of data acquisition and interpretation across devices and contexts, once properly validated in individuals with chronic insomnia [53, 54]. With continued technological refinement and improved data validity, it is conceivable that, in the near future, the combined use of advanced wearable technologies in real-world environment and comprehensive clinical assessment could allow for individualized cardiovascular risk stratification within routine clinical practice based on patients’ phenotypic features and digitally derived metrics for insomnia.

Such paradigm shift in the conceptualization and assessment of insomnia may also provide critical insights into COMISA, a condition characterized by even greater complexity in phenotypic expression and pathophysiological mechanisms. OSA and insomnia are currently considered two coexisting disorders, for which disentangling causal relationships in clinical practice remains challenging, if not impossible. Population-based studies have shown that individuals with OSA are at-risk of developing insomnia symptoms but not necessarily chronic insomnia, which adds further complexity to the issue of COMISA as a constellation of symptoms or disorders [55, 56]. From a theoretical perspective, OSA—with its sleep fragmentation, low arousal threshold, nocturnal awakenings, and hyperactivation of the sympathetic nervous system and HPA axis, could precipitate DMS or EMA, thereby triggering insomnia symptoms [18, 43, 57, 58]. Conversely, the impact of chronic insomnia on the severity and manifestation of OSA is less well described in the literature, with findings remaining inconsistent [18, 57]. The application of emerging technologies, along with data obtained from in-lab and ambulatory PSG or sleep apnea testing, may, however, provide valuable insights and help address some of these unresolved questions.

It is therefore evident that a clearer definition and deeper understanding of these disorders will contribute to the optimization of therapeutic strategies for our patients. Indeed, cognitive behavioral therapy for insomnia (CBT-I) is the first-line, guideline-recommended treatment for insomnia disorder, after widespread misuse of hypnotic and sedative agents in prior decades, given its demonstrated efficacy over sleep hygiene, pharmacotherapy and placebo [59]. CBT-I is an evidenced-based, structured treatment administered by trained clinicians, most typically, although not always, psychologists board-diplomate in behavioral sleep medicine. CBT-I targets the behavioral and cognitive-emotional mechanisms perpetuating insomnia, thereby improving sleep efficiency and reducing cognitive-emotional hyperarousal through behavioral interventions, primarily sleep restriction therapy, and cognitive interventions, such as cognitive restructuring [59]. An interesting clinical observation has shown that the use of phenotypic classification based on objective TST revealed differential treatment responses: the INSD phenotype shows greater responsiveness to CBT-I, whereas the ISSD phenotype appears to benefit more from pharmacotherapy (e.g., trazodone, lemborexant) [60–64]. A plausible explanation for the differential treatment response is that the INSD phenotype is predominantly driven by cognitive-behavioral mechanisms (e.g., cognitive-emotional activation and excessive time in bed), whereas the ISSD phenotype is predominantly driven by physiological mechanisms (e.g., dysregulation of the HPA axis) [14, 19, 61]. This differential treatment effects is expected to be further substantiated by forthcoming randomized controlled trials. Moreover, it would be of considerable interest to investigate the effect of both pharmacotherapy and CBT-I on BP levels and BP dipping in hypertensive as well as normotensive individuals with insomnia disorder, as such findings could elucidate potential therapeutic implications for cardiovascular risk management. Li et al. demonstrated that among hypertensive patients with insomnia, those treated with estazolam exhibited a significant reduction in both systolic and diastolic BP levels compared with those receiving placebo [65]. Surprisingly, despite the widespread use of pharmacotherapy with hypnotic agents such as trazodone in the United States, which has been shown to lower BP and produce orthostatic effects [66, 67], there is a notable lack of observational studies or randomized controlled trials investigating these effects as potentially therapeutic in those with clinical hypertension and insomnia.

Regarding the therapeutic management of patients with COMISA, evidence indicates that they tend to exhibit poorer adherence to CPAP therapy and are more likely to discontinue treatment [18], while there are no clear clinical guidelines for the management of this specific population. However, treatment of insomnia has been shown to improve adherence to OSA therapy [18] and Sweetman et al. showed that CBT-I improves insomnia in COMISA patients independent of their CPAP treatment status [68]. These findings further suggest that addressing insomnia may be key in the management of COMISA as a true comorbidity rather than as a secondary manifestation of one disorder caused by the other. In this context, a phenotype-based approach to insomnia, as previously described for ISSD, could potentially lead to even more tailored and effective therapeutic outcomes. For example, a patient with a COMISA/INSD phenotype may respond more favorably to CBT-I, whereas a COMISA/ISSD phenotype might demonstrate a better response to combination treatment (i.e., CBT-I plus pharmacotherapy). This precision-based framework could ultimately enhance both adherence to respiratory therapy and desired clinical outcomes in patients with COMISA.

## Conclusions

Evidence demonstrates that insomnia is a risk factor for hypertension, with the ISSD phenotype carrying this significant risk. In addition, comorbid insomnia appears to further increase the risk of hypertension already present in patients with OSA (i.e., COMISA) and that the ISSD phenotype also carries most of this risk, further reinforcing findings from studies examining each condition in isolation. This suggests that the coexistence of sleeplessness, short sleep and disordered breathing in COMISA may have additive or synergistic effects on cardiovascular risk, highlighting the importance of assessing and managing insomnia even within populations already diagnosed with OSA and leveraging their objective TST for clinical decision-making. A clear understanding of these concepts and the establishment of a unified framework within the scientific community will facilitate the design of randomized controlled trials aimed at further substantiating these observations. Consequently, this common language will provide the tools necessary for improved diagnostic and therapeutic strategies, advancing patient care in the era of personalized medicine.

## Key References

- Li L, Gan Y, Zhou X, Jiang H, Zhao Y, Tian Q, He Y, Liu Q, Mei Q, Wu C, Lu Z. Insomnia and the risk of hypertension: A meta-analysis of prospective cohort studies. *Sleep Med Rev*. 2021; 56:101403.
  - ○ This meta-analysis examined the association of insomnia symptoms with hypertension in large population-based samples.
- Dai Y, Vgontzas AN, Chen L, Zheng D, Chen B, Fernandez-Mendoza J, Karataraki M, Tang X, Li Y. A meta-analysis of the association between insomnia with objective short sleep duration and risk of hypertension. *Sleep Med Rev*. 2024; 75:101914.
  - ○ This meta-analysis examined the association of insomnia disorder with short sleep duration with hypertension in large population-based and clinical samples.
- Li X, Sotres-Alvarez D, Gallo LC, et al. Associations of sleep-disordered breathing and insomnia with incident hypertension and diabetes. The Hispanic Community Health Study/Study of Latinos. *Am J Respir Crit Care Med*. 2021; 203(3):356–365. doi:10.1164/rccm.201912-2330OC
  - ○ This is one of the first studies to examine the impact of comorbid insomnia and sleep apnea on hypertension in a large population-based sample.

## Data Availability

No datasets were generated or analysed during the current study.

## Abbreviations

AHI: Apnea–hypopnea index
BMI: Body mass index
CBT-I: Cognitive behavioral therapy for insomnia
COMISA: Comorbid insomnia and sleep apnea
DIS: Difficulty initiating sleep
DMS: Difficulty maintaining sleep
ΕΜΑ: Early morning awakening
HPA axis: Hypothalamic–pituitary–adrenal axis
INSD: Insomnia with normal sleep duration
ISSD: Insomnia with short sleep duration
MSLT: Multiple Sleep Latency Test
OSA: Obstructive sleep apnea
PSAC: Penn State Adult Cohort
TST: Total sleep time
